# Transformation of epidermal growth factor receptor T790M mutation‐positive adenosquamous carcinoma of the lung to small cell carcinoma and large‐cell neuroendocrine carcinoma following osimertinib therapy: an autopsy case report

**DOI:** 10.1002/rcr2.402

**Published:** 2019-02-21

**Authors:** Shuhei Moriguchi, Hironori Uruga, Takeshi Fujii, Yoichi Yasunaga, Yui Takahashi, Kazuma Kishi

**Affiliations:** ^1^ Department of Respiratory Medicine Respiratory Center Tokyo Japan; ^2^ Okinaka Memorial Institute for Medical Research Tokyo Japan; ^3^ Department of Pathology Toranomon Hospital Tokyo Japan; ^4^ Department of Pathology, Graduate School of Medicine The University of Tokyo Tokyo Japan

**Keywords:** Epidermal growth factor receptor mutation, large‐cell neuroendocrine carcinoma, osimertinib, small‐cell lung carcinoma, T790M, transformation

## Abstract

A 59‐year‐old man with relapsed epidermal growth factor receptor (*EGFR*) exon 19 deletion‐positive stage IA adenosquamous carcinoma after lobectomy was treated with erlotinib and bevacizumab for 1 year followed by erlotinib alone for 1 year. Because of mediastinal and supraclavicular lymphadenopathy and a nodule on the left anterior chest wall, the patient underwent repeat biopsy from the supraclavicular lymph node. Pathological analysis demonstrated adenosquamous carcinoma harbouring *EGFR* exon 19 deletion and T790M mutation. Osimertinib treatment was therefore started. Six months later, the patient underwent a second‐repeat biopsy from the mediastinal lymph nodes and liver metastases. Both specimens showed small cell lung carcinoma (SCLC). After chemotherapies for SCLC, he died from lung cancer. An autopsy demonstrated tumour heterogeneity, including histological types of adenosquamous, SCLC, and large‐cell neuroendocrine carcinoma. Repeat biopsies at the time of disease progression are useful to choose subsequent treatment for patients with *EGFR* mutation‐positive lung cancer.

## Introduction

Resistance mechanisms to osimertinib are of two types: epidermal growth factor receptor (*EGFR*) dependent, such as point mutations containing C797S, and *EGFR* independent, such as bypass signal pathway or histological transformation to small‐cell lung carcinoma (SCLC) and large‐cell neuroendocrine carcinoma (LCNEC)) 1, 2. Here, we report an autopsy case with *EGFR* T790M‐positive adenosquamous carcinoma that transformed to *EGFR* T790M‐negative SCLC and LCNEC after osimertinib therapy.

## Case Report

**Figure 1:**
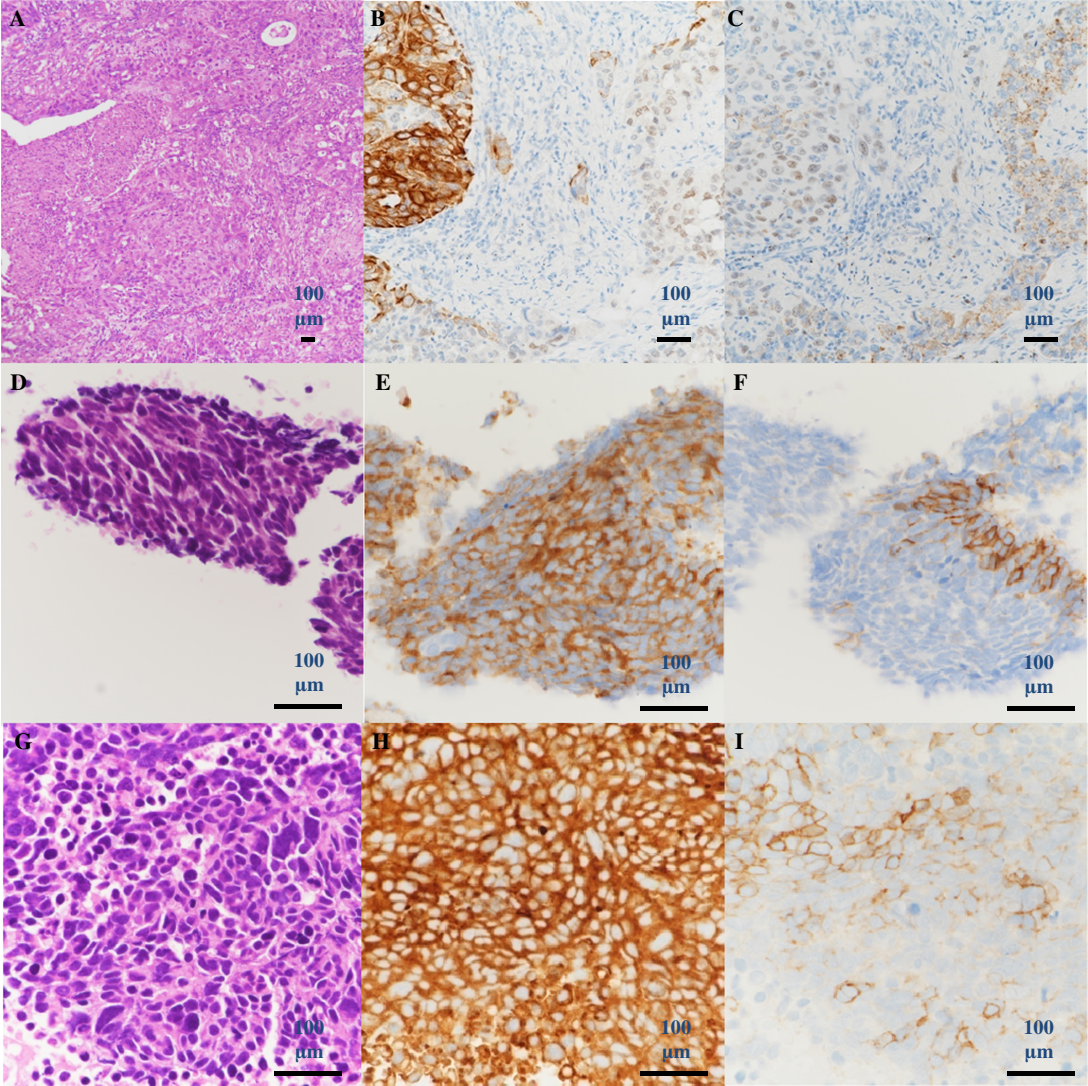
Histology of lung surgical specimen (A–C); (A) haematoxylin and eosin, (B) TTF‐1 and CK5/6, and (C) p40 and napsin stains. Histology of endobronchial ultrasound‐guided transbronchial needle aspiration specimens from mediastinal lymph nodes (D–F); (D) Haematoxylin and eosin, (E) synaptophysin, and (F) CD56 stains. Histology of liver biopsy (G–I), (G) Haematoxylin and eosin, (H) synaptophysin, and (I) CD56 stains.

A 59‐year‐old man underwent video‐assisted thoracic surgery of the left upper lobe with ND2a‐1 lymphadenectomy 4 years prior to disease presentation and was diagnosed as having stage IA (T1bN0M0) adenosquamous carcinoma of the lung (Fig. 1A–C) harbouring *EGFR* exon 19 deletion based on the American Joint Committee on Cancer staging system, seventh edition. He was a non‐smoker with no previous medical problems. Two years after the surgery, he was diagnosed as having recurrence in the mediastinal lymph nodes and left anterior chest wall. Erlotinib (150 mg once daily) and bevacizumab (15 mg/kg every 3 weeks) were started as first‐line therapy. After 19 months of the therapy, the lung cancer also metastasized to the right supraclavicular lymph node. He underwent percutaneous supraclavicular lymph node needle biopsy as the first‐repeat biopsy. Adenosquamous carcinoma harbouring *EGFR* exon 19 deletion and T790M mutation was detected. Osimertinib (80 mg once daily) was started, and the patient achieved partial response. Seven months after starting treatment with osimertinib, the mediastinal lymphadenopathy recurred, and multiple new liver metastases were seen. The second‐repeat biopsy for the subcarinal mediastinal lymph nodes was performed. The histological diagnosis was SCLC harbouring *EGFR* exon 19 deletion without T790M mutation (Fig. 1D–F). He was treated with combination chemotherapy of cisplatin (80 mg/m^2^, day 1, every 3 weeks) and etoposide (100 mg/m^2^, days 1–3, every 3 weeks). After one cycle of chemotherapy, computed tomography (CT) imaging demonstrated that the mediastinal lymph nodes had shrunk, but the hepatic metastases had disseminated. Fine‐needle aspiration biopsy of the liver was performed, and the histological diagnosis was also SCLC harbouring *EGFR* exon 19 deletion without T790M mutation (Fig. 1G–I). After four cycles of chemotherapy, the size of the mediastinal lymph nodes further decreased, but the hepatic metastases increased.

**Figure 2:**
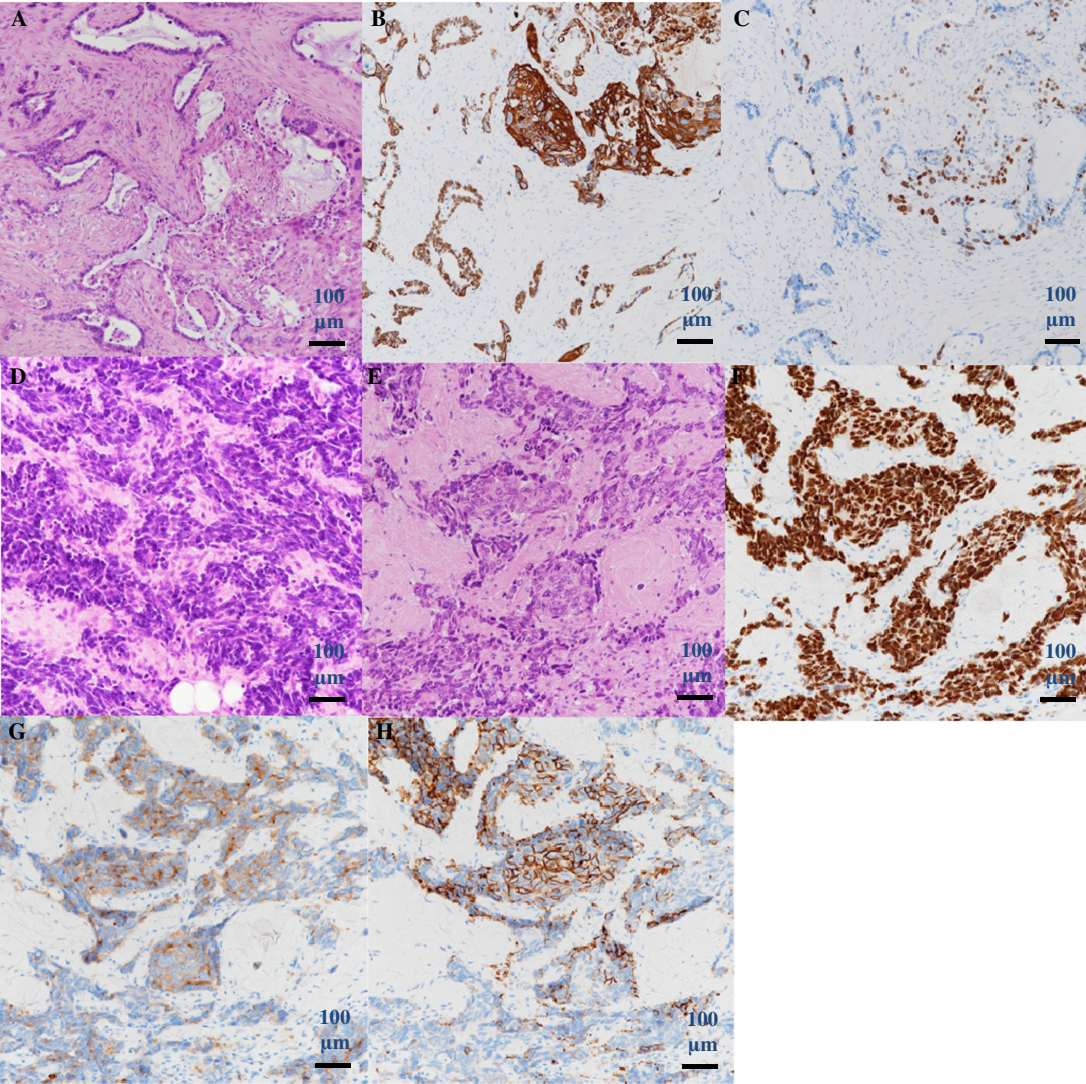
Histology of autopsy (A–H); (A) haematoxylin and eosin staining of adenosquamous carcinoma component, (B) TTF‐1 and CK5/6 stains, (C) p40 and napsin stains, (D) haematoxylin and eosin staining of small cell carcinoma component, (E) haematoxylin and eosin staining of large‐cell neuroendocrine carcinoma component, (F) TTF‐1 stain, (G) synaptophysin stain, and (H) CD56 stain.

Two months later, liver metastases became worse, and new lesions of the bone metastases at spine developed. We administered amrubicin (35 mg/m^2^, 75% on days 1–3, every 3 weeks) for the next treatment. However, the treatment was changed to irinotecan (100 mg/m^2^, 75% on day 1, 8, and 15 every 4 weeks) after one cycle of amrubicin because of elevated liver enzymes and pancytopenia. After two cycles of irinotecan, he developed superior vena cava syndrome, which was treated with radiotherapy. He was died 13 months after diagnosis of transformation to SCLC. An autopsy was performed, and microscopic examination showed metastases to lungs, mediastinum, cervical lymph nodes, liver, left kidney, right adrenal gland, bones, and epicardium. Microscopic examination demonstrated tumour heterogeneity of various organs. Although histological types of most metastatic lesions were SCLC or LCNEC, adenocarcinoma was found in the left kidney, coexistence of adenosquamous carcinoma and SCLC was observed in mediastinum, and adenocarcinoma and SCLC were found in right adrenal gland and cervical lymph nodes (Fig. 2). LCNEC of epicardium also showed *EGFR* exon 19 deletion without T790M mutation.

## Discussion

We report a patient with *EGFR* T790M‐positive adenosquamous carcinoma that transformed to *EGFR* T790M‐negative SCLC and LCNEC after treatment with osimertinib. *EGFR* exon 19 deletion was detected in both adenosquamous carcinoma and SCLC.

SCLC transformation following treatment with first‐and second‐generation EGFR‐tyrosine kinase inhibitors (TKIs) has been reported in 3–15% of patients 2, 3. As in cases of adenocarcinoma changing to SCLC, the inactivation of retinoblastoma 1 (*RB1*) and tumour protein 53 (*TP53*) were also reported 4, 5.

SCLC transformation due to osimertinib is rare, and its frequency is still unknown. To our knowledge, only nine cases of SCLC transformation after osimertinib treatment have been reported 6, 7, 8, 9, 10 (Table 1).

**Table 1:** Previous reports with transformation of non‐small‐cell lung adenocarcinoma to small‐cell lung carcinoma (SCLC) and large‐cell neuroendocrine carcinoma (LCNEC) following osimertinib therapy.

		Gender	Age	Stage	Histology at diagnosis	EGFR status at diagnosis	EGFR‐TKIs before osimertinib	EGFR status after first re‐biopsy	Detection	Best response with osimertinib	Response duration (months)	Histology after second re‐biopsy	EGFR status after second re‐biopsy	Treatment
1	Kim et al. 6	Female	57	ND	Ad	19 del	Gefitinib	19 del + T790M +	Tissue	PR	11	SCLC	19 del + T790M −	Radiation CBDCA+VP16
2	Ham et al. 7	Female	57	cT3N2M1a IVa	Ad	L858R	Erlotinib	L858R + T790M +	Tissue	PR	14	SCLC	L858R + T790M − EGFR amp	CBDCA+VP16
3	Ham et al. 7	Female	58	Reccurent	Ad	Wild	Erlotinib Afatinib	19 del + T790M +	Tissue	PR	18	SCLC	19 del + T790M −	CBDCA+VP16
4	Li et al. 8	Female	52	Reccurent	Ad	19 del	Erlotinib	19 del − T790M −	Plasma	PR	6	SCLC	19 del + T790M −	CBDCA+VP16 +osimertinib
5	Minari et al. 9	Male	69	ND	Ad	19 del	Afatinib	19 del + T790M +	Plasma	PD	3	SCLC	19 del − T790M −	BSC
6	Minari et al. 9	Female	70	ND	Ad	19 del	Afatinib	19 del + T790M +	Plasma	PD	2	SCLC	19 del + T790M −	BSC
7	Minari et al. 9	Male	55	ND	Ad	19 del	Gefitinib	19 del + T790M +	Plasma	PD	3	SCLC	19 del + T790M −	CBDCA+VP16
8	Minari et al. 9	Male	68	ND	Ad	L858R	Afatinib	L858R + T790M +	Plasma	PD	3	SCLC	L858R + T790M −	CBDCA+VP16
9	Taniguchi et al. 10	Female	67	Reccurent	Ad	19 del	Gefitinib Erlotinib	L858R + T790M +	Tissue	ND	13	SCLC	19 del + T790M −	CBDCA+VP16
10	Baglivo S, et al. 11	Male	57	cT1bN2Mab IVb	Ad	19 del	Erlotinib	19 del + T790M +	Tissue	1	PD	LCNEC	19 del + T790M −	Radiation Adrrenalectomy CDDP+VP16
11	Ricordel et al. 12	Male	57	cT3N2M1b IVb	Ad	19 del	Gefitinib	19 del + T790M +	Tissue	SD	10	LCNEC	19 del + T790M +	Radiation Osimertinib
	This case	Male	63	Reccurent	Ad‐Sq	19 del	Erlotinib+Bev	19 del + T790M +	Tissue	PR	7	SCLC and LCNEC	19 del + T790M −	CDDP+VP16

19 del, exon 19 deletion; Ad, adenocarcinoma; Ad‐Sq, adenosquamous cell carcinoma; BSC, best supportive care; CBDCA, carboplatin; CDDP, cisplatin; L858R, L858R point mutation; ND, not described; PD, progressive disease; PR, partial response; SD, stable disease; T790M, T790M point mutation; VP16, etoposide.

In the autopsy findings of this case, LCNEC was also found along with SCLC. Several cases with transformation from adenocarcinoma to LCNEC were reported as a resistant mechanism to the first‐ and second‐generation EGFR‐TKIs along with two cases to osimertinib (Table 1) 11, 12. LCNEC was a very heterogeneous group. A next‐generation sequencing (NGS) study of LCNEC from the Memorial Sloan Kettering Cancer Center showed that 18 of 45 LCNEC cases had SCLC‐like molecular profile, but 25 had non‐small cell lung carcinoma (NSCLC)‐like molecular profile 13. These data made sense to us because LCNEC coexisted in our case, transforming from adenosquamous carcinoma to SCLC.

Several studies showed heterogeneity of acquired resistance to EGFR‐TKIs. Because the heterogeneity of expression of immune markers depends on the metastatic sites and histological transformation, and the biopsy of one lesion may not represent the immune marker status of all lesions 14. In this case, we conducted second‐repeat biopsies from two metastatic sites (mediastinal lymph nodes and liver), which resulted in an observation of SCLC with *EGFR* exon 19 deletion without T790M mutation. However, the results on both biopsies were a little different from autopsy findings, a strange mix of SCLC, LCNEC, and adenocarcinoma. This result showed limitation of small biopsies and usefulness of repeated biopsies from different sites to accumulate information for a clinical decision.

Identifying the histological diagnosis and driver mutations is important in deciding treatment options. Given the various mechanisms of acquired resistance, repeat biopsy at the time of disease progression is useful to guide the direction of subsequent treatment in patients with *EGFR* mutation‐positive lung cancer.

### Disclosure Statement

Appropriate written informed consent was obtained for publication of this case report and accompanying images.
